# The effect of the COACH program (Continuity Of Appropriate pharmacotherapy, patient Counselling and information transfer in Healthcare) on readmission rates in a multicultural population of internal medicine patients

**DOI:** 10.1186/1472-6963-10-39

**Published:** 2010-02-16

**Authors:** Fatma Karapinar-Çarkıt, Sander D Borgsteede, Jan Zoer, Carl Siegert, Maurits van Tulder, Antoine CG Egberts, Patricia MLA van den Bemt

**Affiliations:** 1 Department of Hospital Pharmacy, Amsterdam, The Netherlands; 2 Division of Pharmacoepidemiology and Pharmacotherapy, Faculty of Science, Utrecht University, Utrecht, The Netherlands; 3 Netherlands Pharmacovigilance Centre Lareb, 's-Hertogenbosch, The Netherlands; 4 Department of Internal Medicine, Amsterdam, The Netherlands; 5 Department of Health Sciences, Faculty of Earth & Life Sciences, Amsterdam, The Netherlands; 6 Department of Clinical Pharmacy, University Medical Centre Utrecht, Utrecht, The Netherlands; 7 Department of Hospital Pharmacy, PO Box 2040, 3000 CA, Rotterdam, The Netherlands

## Abstract

**Background:**

Medication errors occur frequently at points of transition in care. The key problems causing these medication errors are: incomplete and inappropriate medication reconciliation at hospital discharge (partly arising from inadequate medication reconciliation at admission), insufficient patient information (especially within a multicultural patient population) and insufficient communication to the next health care provider. Whether interventions aimed at the combination of these aspects indeed result in less discontinuity and associated harm is uncertain. Therefore the main objective of this study is to determine the effect of the COACH program (Continuity Of Appropriate pharmacotherapy, patient Counselling and information transfer in Healthcare) on readmission rates in patients discharged from the internal medicine department.

**Methods/Design:**

An experimental study is performed at the internal medicine ward of a general teaching hospital in Amsterdam, which serves a multicultural population. In this study the effects of the COACH program is compared with usual care using a pre-post study design. All patients being admitted with at least one prescribed drug intended for chronic use are included in the study unless they meet one of the following exclusion criteria: no informed consent, no medication intended for chronic use prescribed at discharge, death, transfer to another ward or hospital, discharge within 24 hours or out of office hours, discharge to a nursing home and no possibility to counsel the patient.

The intervention consists of medication reconciliation, patient counselling and communication between the hospital and primary care healthcare providers.

The following outcomes are measured: the primary outcome readmissions within six months after discharge and the secondary outcomes number of interventions, adherence, patient's attitude towards medicines, patient's satisfaction with medication information, costs, quality of life and finally satisfaction of general practitioners and community pharmacists.

Interrupted time series analysis is used for data-analysis of the primary outcome. Descriptive statistics is performed for the secondary outcomes. An economic evaluation is performed according to the intention-to-treat principle.

**Discussion:**

This study will be able to evaluate the clinical and cost impact of a comprehensive program on continuity of care and associated patient safety.

**Trial registration:**

Dutch trial register: NTR1519

## Background

Medication errors are the most common type of errors affecting patient safety, occurring most frequently at points of transition in care [1-3]. There are three key problems causing these medication errors at hospital admission and discharge. The first problem is incomplete and inappropriate medication lists. This problem starts at hospital admission for example due to recall bias of the patient, incomplete medication records (e.g. absence of over-the-counter drugs) and inappropriately prescribed drugs (e.g. indication of pre-hospital prescribed drugs not evaluated) [4]. These admission medication errors can carry over to the discharge medication and new medication errors can occur for example when hospital physicians forget to restart temporarily discontinued medication or do not evaluate the appropriateness of discharge medication [5,6].

The second problem is insufficient patient information. While receiving care in hospital patients often get help with the preparing and administering of their medication by hospital staff. However, following hospital discharge patients are abruptly expected to manage their medication themselves, with little support or preparation [3].

The last problem regards insufficient communication to the next health care provider. Discharge letters and discharge prescriptions generally do not contain the entire pharmacotherapy [7,8]. This incompleteness could lead to confusion about whether the medication which is not listed is discontinued or just not mentioned. Both the general practitioner and community pharmacy are not informed on reasons for changes in the pharmacotherapy leading to confusion whether these changes should be maintained or were temporal [9,10].

Evidence exists on the effect of discharge medication related interventions on reducing adverse events, reducing the readmission rate and improving adherence [5,11-15]. However, some studies showed no effect and Holland et al. reported contradictory results on readmission rates [16-18]. Most studies have not combined intervention types to solve the problems as described above. For example, medication reconciliation is often performed with the use of medication records without active involvement of patients, or in case patients are involved, patients who are unable to speak the native language of the study location are excluded [12,16,19-21]. In contrary, some studies are so comprehensive that it is expected that most hospitals cannot implement such time consuming interventions (e.g. one study reports 2 hours per patient) [21-23]. Furthermore, in general the intervention is performed (partly) by pharmacists making the intervention expensive [5,11-19,21-23]. It is unknown what the effect is of discharge medication related interventions when they are performed by healthcare providers with a lower level of education.

Therefore, the COACH (Continuity Of Appropriate pharmacotherapy, patient Counselling and information transfer in Healthcare) program is designed to improve continuity of care by combining interventions, including non-native patients and using pharmaceutical consultants (i.e. pharmacy technicians who have followed additional training) to perform the intervention. The intervention consists of medication reconciliation at discharge (in addition to medication reconciliation at admission to prevent medication errors from carrying over to the discharge medication), patient counselling at discharge and communication of medication information to the next healthcare providers.

At present it is unknown whether such an intervention program indeed can lead to less discontinuity and associated patient harm. Therefore, the main objective of this study is to determine the effect of the COACH program on readmissions after six months in a multicultural population from the internal medicine department.

## Methods/Design

### Design

**Figure 1:**
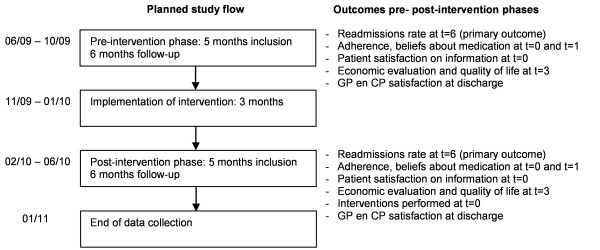
**Study flow of the COACH program**. t = 0,1,3,6; at hospital discharge, one month, three months, six months after hospital discharge. GP = general practitioner. CP = community pharmacist.

A prospective experimental study with a before-after design is performed at the St. Lucas Andreas Hospital in the Netherlands, a 550-bed general teaching hospital serving a multicultural population. The study is carried out from June 2009 through Januari 2011. The effects between a usual care group and an intervention group (pre- and post-intervention measurement design) are compared. First, patients are included during five months in the usual care group (pre-intervention phase with six months follow-up). Second, the intervention is implemented in the study ward (implementation phase of 3 months). Finally, patients are included during five months in the intervention group (post-intervention phase with six month follow-up, see figure 1 for flowchart and measurements).

### Study population

The study is performed at the internal medicine ward. All patients admitted with at least one prescribed drug intended for chronic use are invited to participate. Exclusion criteria are: no informed consent, no medication intended for chronic use prescribed at discharge, death, transfer to another ward or hospital, discharge within 24 hours or out of office hours, discharge to a nursing home (as patients do not administer their own medication) and patients who cannot be counselled (as stated by the resident due to physical/mental constraints, being critically ill or due to language restrictions without relatives or healthcare personnel to translate, i.e. languages other than Dutch, Turkish, English and Arabic/Berber). Only the patient's first hospital admission is included in the study period (readmissions are the main outcome measure). The St. Lucas Andreas Hospital institutional review board has stated that this study is exempt from review by the institutional review board as the Dutch legislation does not request this for studies that do not affect the patient's integrity. In this study the burden was considered minimal for the patient and therefore the medical ethics committee waived the review. The burden is minimal as the patient will receive counselling about his discharge medication in the intervention group. This should be usual practice as the hospital has a legal obligation to inform patients. The patients is also asked to fill in questionnaires and cost diaries. This is expected to take 60 minutes of the patient's time. To respect the wish of a patient to participate in a study we decided to ask the patients for an informed consent to obtain information from their general practitioner on readmission rates and for filling in the questionnaires/cost diaries. Patient data are sampled and stored in accordance with privacy regulations.

### Study procedures

#### Usual care

##### Medication reconciliation on admission

At hospital admission residents mostly use the information provided by patients (or relatives) or previous hospital records (e.g. discharge letters, patient charts) to examine the pre-admission medication. However, medication reconciliation is not structurally performed by the resident. Residents can consult the medication records of the community pharmacy through a link in the hospital's Computerized Physician Order Entry (CPOE) system for patients that are within the catchment area of the hospital. If a community pharmacy is not connected to the hospital's CPOE, the resident can request the hospital pharmacy to obtain a faxed medication list from the community pharmacist. The resident registers the admission prescriptions in the hospital's CPOE where after the prescriptions are checked during hospital admission by the clinical pharmacist on dosages, double medication, drug-drug interactions and contra-indications.

At present information on allergies is not structurally provided to the clinical pharmacist making medication surveillance on allergies impossible.

##### Medication reconciliation at discharge

The resident prints a medication list from the hospital's CPOE. On this medication list the resident can adjust the medication and he then indicates which medication should be dispensed by the community pharmacy. The medication list is sent to the hospital pharmacy. The pharmacy technician screens the list for obvious errors (e.g. dose not provided) but no structured medication reconciliation is performed.

##### Patient counselling at discharge

To support the patient counselling a medication list is written down by the resident using the information in the hospital's CPOE. At present, residents and nurses are involved in patient instructions on pharmacotherapy. For both professionals this aspect is only a relatively small part of a large amount of tasks, making the time to be spent on medication related patient instructions rather limited or the patient counselling is not performed at all. Also, the knowledge necessary for providing adequate instructions is often insufficient in residents (inexperienced) and nurses (training provides little knowledge on drugs).

##### Communication of discharge medication

After screening of the medication list by the pharmacy technician at discharge, the discharge prescriptions are sent to the community pharmacy. The community pharmacist is mostly informed on medication which should be dispensed. The reasons for changes in therapy or clinical information such as allergies are not provided.

The communication to the general practitioner takes place through the discharge letter in which the medication is typed by the resident. The medication list in the discharge letter is generally incomplete and provides little or no information on changes in the pharmacotherapy and the reasons for these changes.

### COACH intervention program

The COACH intervention program is carried out by a team of pharmaceutical consultants with clinical pharmacists as supervisors. Pharmaceutical consultants are pharmacy technicians who have followed an additional three year bachelor program which is focused on pharmaceutical patient care. They are specifically trained in pharmacotherapy and communication with patients. In contrast to nurses and residents, they can dedicate more time to the patient, as this job is their main task. Because of their lower level of education, when compared to pharmacists who have had a 6 year university training, salary expenditures for pharmaceutical consultants are lower, which is why they are used besides a supervising pharmacist.

As 30-40% of the patient population in the St. Lucas Andreas hospital is originating from foreign countries (migrants, mostly Turkish and Moroccan) information leaflets and questionnaires are available in Dutch, Turkish, Arabic and English. Arabic is the written language of Morocco, but Moroccan immigrants in the Netherlands often use the Berber language (a non-written language) and are unable to read Arabic. In those cases relatives or healthcare personnel are asked to translate the information leaflets.

**Figure 2:**
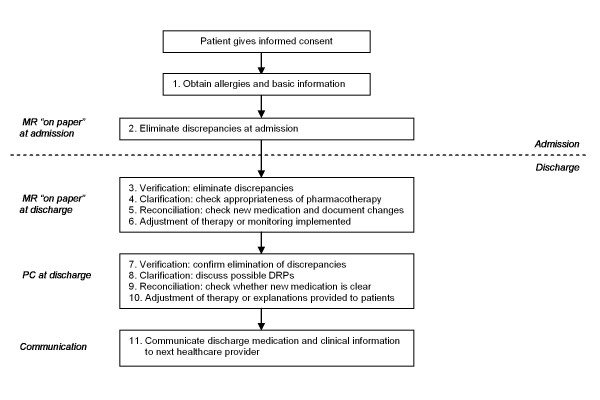
**Implementation of the COACH program**. MR = medication reconciliation. PC = patient counselling following the steps for medication reconciliation. DRPs = drug related problems.

The COACH program consists of four main processes that are subdivided in subprocesses (see figure 2). Although the program focuses on discharge, a small part of the intervention is carried out on admission to prevent admission medication errors to carry over to the discharge medication.

#### Obtaining basic information and medication reconciliation on admission

After the pharmaceutical consultant gets informed consent from the patient at admission the consultant asks the patient about possible allergies. If any are mentioned the pharmaceutical consultant registers the allergy in the CPOE for medication surveillance purposes. Furthermore, the language spoken by the patient is checked. If a patient cannot speak or understand Dutch a family member or friend is asked to be present and translate or a specific health care worker speaking the native language of the patient is added to the team. Finally, the pharmaceutical consultant asks some additional basic information (see figure 2 and table 1, step 1). Hereafter an information leaflet is given to the patient. This leaflet informs the patient further about the project and motivates the patients to ask questions about medication during patient counselling at discharge.

**Table 1:** Protocols used for the steps shown in figure 2.

Steps	Protocols used consists of
1	Questions asked: allergies, presence of relative during patient counselling at discharge, marital status, birth country patient and parents, education, readmission rate previous six months

2	Check: - Matching of medication at admission with pre-admission medication regarding drug, dose, route and frequency

3	Check: - Matching of medication at discharge with pre-admission medication regarding drug, dose, route and frequency - Whether temporally discontinued medication and substituted medication (due to hospital formulary policy) should be resumed

4	Check: - Continuing need: discontinue not indicated (temporally prescribed) medication - Consider right dose (e.g. for geriatric patient), simplify drug regimen (e.g. modified release product in stead of plain drug), duration of therapy (e.g. antibiotic prescribed too long, gradually reduce prednisolone) - Laboratory values: international normalized ratio, glomerular filtration rate, glucose, sodium and potassium blood levels to adjust medication if necessary. - Identify suboptimal treatment (e.g. laxative with opioid, gastroprotection with NSAID and risk factors, rescue medication with inhaled corticosteroid, bisfosfonate with long-term prednisolone, isordil with ACS, statin with diabetes mellitus type II) - Drug-drug interactions (pharmacokinetic and pharmacodynamic) and contra-indications (e.g. NSAID with heart failure, COX-2 inhibitor with ischemic heart disease) - Consider cost (e.g. brand to generic drug) - Consider monitoring (e.g. therapeutic drug monitoring, electrolytes, creatin)

5	Check: - Appropriateness of new medication - Documentation of (reasons for) changes between discharge prescriptions and pre-admission medications

7	Check: - How medication is used by the patient and at what time point. - Continuing need: discontinue not indicated (temporally prescribed) medication or restart medication if patient does not agree with discontinuation (e.g. patient still has pain) - Other medication usage (e.g. over-the-counter medication or herbals) to evaluate whether there are contra-indications or interactions with the medication prescribed at discharge

8	Check: - Practical problems with medication use: check whether patient is capable of using his medication (e.g. big tablets, type of inhalator) - Occurrence of adverse drug reactions: check whether these could be prevented or minimized - Forgetting of medication: check whether patient is compliant and what the possible reasons are for non-adherence. Problems with adherence are further explored and possible tools, such as pill boxes, are discussed.

9	Check: - Understanding of new prescribed medication - Knowledge of side effects (e.g. bloody or black tarry stools with anticoagulants to recognize bleeding, risk of fracture and prednisolone, increase of blood sugar and prednisolone, rapid heart beats and bronchodilators, sore throat and inhalalation corticosteriods to rinse mouth, stomach pain and NSAID, headache and nitrates/beta-blockers, muscle pain and lipid-lowering medicines, orthostatic hypotension and antihypertensives, diarrhoea and antibiotics, risk of falls/drowsiness and hypnotics, muscle weakness and paraesthesia to recognize low/high potassium) - Written information need: give patient information leaflet for new prescribed medication - Whether there are questions - Which medication the patient still has in stock at home and which medication should be dispensed.

11	Register on the medication discharge overview: changes in medication and reasons, possible drug-related problems and follow-up procedures (e.g. therapeutic drug monitoring). This information is automatically registered on the medication summary for the patient also.

ACS = Acute coronary syndrome

After the resident registers the admission prescription in the hospital's CPOE the pharmaceutical consultant verifies these prescriptions using community pharmacy records without counselling the patient (due to time constraints and because the resident already counsels the patient following routine care). All discrepancies ("on paper") with the pre-admission medication, known allergies and possible drug-related problems are communicated to the resident with a standardized form (see figure 2 and table 1, step 2). The resident can adjust the prescriptions if necessary.

#### Medication reconciliation at discharge

At discharge medication reconciliation is performed again using a protocol which contains the steps for medication reconciliation (see figure 2 and table 1, step 3-5) [24]. First in the verification step the presence of discrepancies with the pre-admission medication is examined again by using the medication history of the community pharmacy. Second, in the clarification step the appropriateness of the pharmacotherapy is checked and the pharmacotherapy is evaluated. Also the international normalized ratio, glomerular filtration rate, glucose, sodium and potassium blood levels are checked to adjust medication if necessary. In the third step the newly initiated medication is evaluated to ensure all changes are intentional and changes in the pharmacotherapy are documented. Finally, the results of all steps are discussed with the resident and the prescriptions are adjusted if necessary.

#### Patient counselling at discharge

**Figure 3:**
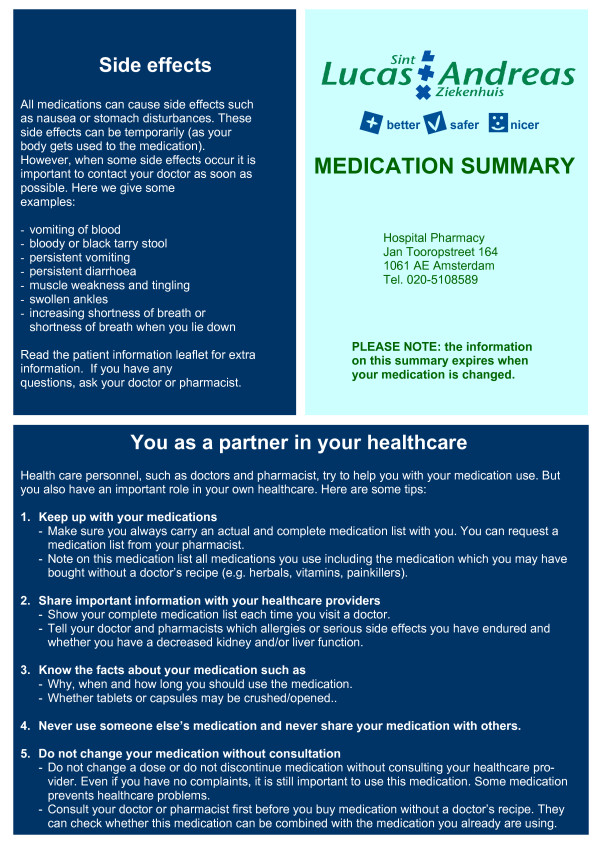
**Medication summary for the patient (reverse side)**. The medication summary is folded to a A6-format.

**Figure 4:**
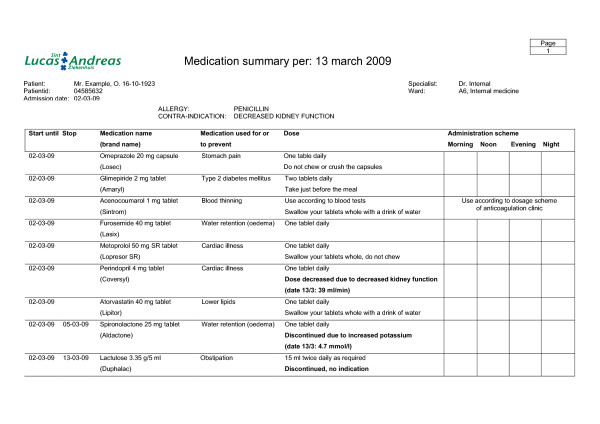
**Medication summary for the patient (front side)**. The administration scheme is filled in with the help of the patient. In bold text the reasons for changes in the pharmacotherapy, drug related problems and follow-up actions can be specified.

To support patient counselling a comprehensive medication summary for the patient is developed (see figure 3 and 4). This double sided medication summary is printed from the hospital's CPOE. One side contains contact information of the hospital, advices on side effects and advices on patient involvement in healthcare (see figure 3). This information is based on several literature reports [25-27]. The other side contains patient data, clinical information (e.g. allergies, contra-indications), hospital physician information, start and stop date for medication, medication name (brand and generic for the patient to recognize his medication), dose information and advices, reason for changes in pharmacotherapy (bold text) and a daily time table (see figure 4).

After the medication reconciliation has been performed and discussed with the resident, the pharmaceutical consultant counsels the patient and/or his family. The patient counselling is also carried out by following the steps for medication reconciliation (see figure 2 and table 1, step 7-9). First, details of all medications are confirmed in the verification step by using the medication summary. The pharmaceutical consultant asks the patient how medication is used (to see whether the use is correct), whether medication is not in use anymore or whether additional medication is used. Second, the clarification step is performed through checking whether improvements can be made on safety and quality of pharmacotherapy and explaining or answering questions. Third, in the reconciliation step new medication is discussed to evaluate whether the patient understands why this medication is prescribed.

The counselling is not only aimed at gathering information about the actual medication usage but also at educating the patient about changes in the pharmacotherapy and involving the patient in the optimisation of the medication usage. If relevant, special attention is paid to subjects relevant for specific patient populations, such as use of medication during fasting. The counselling is inside the hospital, at bedside or in a separate room if preferred by the patient.

The results of patient counselling are discussed with the resident and the prescriptions can be adjusted if necessary. This results in the final discharge medication.

#### Communication of discharge medication

**Figure 5:**
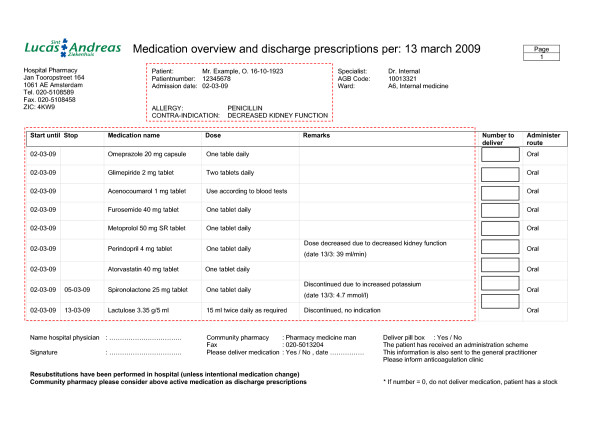
**Discharge medication overview for community pharmacist and general practitioner**. In the remarks section reasons for changes in the pharmacotherapy, drug-related problems and follow-up actions can be specified. This overview is faxed to the community pharmacist. If Vitamin K antagonists are prescribed the text "Please inform anticoagulation clinic" is printed to request the community pharmacy to inform the anticoagulation clinic about the final discharge prescriptions. The information in the red blocks is mailed to the general practitioner.

To support the communication of discharge medication a discharge medication overview is developed (see figure 5). This discharge medication overview is printed from the hospital's CPOE and contains contact information of the hospital, patient data, clinical information (e.g. allergies, contra-indications), hospital physician information, start and stop date of medication, generic medication name, dose information, reasons for changes in pharmacotherapy, drug-related problems, follow-up actions, the amount of medication that has to be dispensed by the community pharmacy, information on tools for adherence and preference of patient to have his medication delivered at home by the pharmacy.

The discharge medication overview is faxed to the community pharmacy before discharge. The overview is also send to the general practitioner by e-mail.

### Study endpoints and data collection

The primary outcome of this study is the readmission rate within six months after discharge. In addition, several secondary outcome measures with respect to medication safety are measured and analysed: number of interventions, adherence, patient's attitude towards medicines and satisfaction with medication information, costs-effectiveness of the intervention, quality of life and satisfaction of general practitioners and community pharmacists.

For collection of outcome parameters, hospital patient records, primary care patient records and validated questionnaires/forms are used. Data are collected prospectively during the pre-intervention and post-intervention period, and in the period up to six months after discharge. The following parameters are registered:

➢ readmission within six months after discharge (primary outcome): the hospital information system is used to register readmissions of the patients in the same hospital. The patient's general practitioner is asked for readmissions in other hospitals.

➢ patient characteristics: these are extracted from the medical records of the hospital information system including gender, age, morbidities, length of stay.

➢ interventions performed in the discharge intervention process: prescribed medication at discharge is extracted from the initial medication order forms in the hospital's CPOE. All changes (due to correction of medication errors or optimisation of pharmacotherapy) in these initial medication orders are registered by the research pharmacist. Also all explanations provided to the patient during patient counselling are registered as an intervention. Interventions performed in the admission process are not documented for this study. The interventions at discharge are classified according to our previously described classification system [28].

➢ patients are asked to fill out a questionnaire about their adherence to drug treatment (MARS; Medication Adherence Rating Scale) [29], satisfaction with information about medicines (SIMS) [30] and their attitude towards drugs (BMQ; Beliefs about Medicines Questionnaire) [31]. After the discharge counselling the patient is given a questionnaire (MARS, BMQ, SIMS) which is filled out before discharge. After one month a second short questionnaire (MARS, BMQ) is sent to evaluate whether adherence and the beliefs about medication have changed after one month.

➢ satisfaction of GPs and community pharmacies: a questionnaire is sent to the patient's general practitioner and community pharmacist within two days of discharge to evaluate their satisfaction with the information on the patient's discharge medication.

➢ cost-effectiveness estimate and quality of life: the aim of the economic evaluation is to determine and compare the total costs of the COACH program compared with usual care in patients and to relate these costs to the effects of these two approaches. The pharmacist and counsellors register the time and material spent on the intervention.

All patients are asked to collect data about healthcare utilisation and quality of life (EuroQol) through monthly sent cost diaries (up to three months) [32]. These cost diaries have been proven to be valid and reliable and have previously been used in economic evaluations in primary care that included patients of Moroccan and Turkish origin [33-38]. The cost diaries are translated and supplied in the patient's preferred language. Healthcare costs, patient and family costs, and production losses are included. All costs of healthcare are assessed as it is hard to distinguish which costs are related to medication use. Healthcare costs include the costs of visits to the general practitioner, medical specialist, hospitalisations and medication costs. Patient and family costs include costs of over the counter medication, informal care and alternative treatments. Costs of productivity losses due to the absence from paid and unpaid work are also estimated.

### Sample size

The primary outcome measure is the readmission rate within six months after discharge. The effects of previous studies into pharmacist pre-discharge medication reconciliation combined with patient counselling on the reduction of the frequency of readmission vary widely [12,13,15,21-23]. Four studies report an absolute decreased readmission rate of 13-30% and two studies report 5-9% (median 15%). Based on a conservative interpretation of these studies, it is estimated that the intervention reduces the proportion of readmitted patients in a comparable population with 10% from 25% in the usual care group to 15% in the intervention group. However, the populations in these studies are not fully comparable to our population: previous studies were limited to elderly patients and our study also includes younger patients. We expect a lower proportion of readmitted patients in both the usual care as the intervention group, because hospital admissions related to medication are less frequent in younger patients compared to elderly, and this probably also applies to hospital readmissions after discharge. As there are no exact numbers for the proportion of readmissions in younger patients, we use the most conservative approach that no patients younger than 65 will be readmitted. At the internal ward in our hospital, the proportion of patients younger than 65 years being discharged is about 20%. Given the assumption that no younger patients are readmitted, the proportion of readmitted patients is 20% lower in both groups. The estimated proportions of readmitted patients are 20% in the usual care and 12% in the intervention group. With these proportions, the expected reduction of readmitted patients is 8%. With a type 1 error of 0.05, a power of 80%, and equal sample sizes, a total of 360 patients per group is needed.

At the Department of Internal Medicine 150-180 patients are being discharged each month. With an estimated proportion of 40% of the patients being excluded due to the exclusion criteria and considering loss to follow-up, it is expected that the period to evaluate usual care and the intervention will take about five months for each group.

Subjects can leave the study at any time for any reason if they wish to do so without any consequences. The number of excluded patients and reasons for exclusion are registered. The same applies to patients who drop out of the study after inclusion. If the agreement with informed consent is not withdrawn, data that have been collected until drop out are included in the analysis.

### Data analysis

Patients from the intervention and control group are compared for all baseline characteristics using relative risks with 95% confidence intervals. For the primary endpoint (readmissions) interrupted time series analysis is used for data-analysis. Baseline data are collected over 5 months (with 3 separate measurements), as will be the post-intervention data. The study design thus meets the criteria for a robust interrupted time series analysis, that is 3 data-points pre- and post-intervention, each consisting of at least 30 patients [39]. Subgroup analysis is performed for ethnicity and the results are corrected for potential confounders such as gender, age and underlying disease. Descriptive statistics is performed for the secondary outcomes (interventions registered, adherence, patient's attitude towards medicines, satisfaction with medication information and satisfaction of the general practitioner and community pharmacist). Continuous measures are summarized using means and standard deviations and categorical measures are summarized using percentages.

The economic evaluation is performed according to the intention-to-treat principle and from a societal perspective. Bootstrapping is used for pair-wise comparison of the mean differences in total costs between treatment groups. Confidence intervals are obtained by bias corrected and accelerated bootstrapping, using 5000 replications. Both a cost-effectiveness and cost-utility analysis is performed. Cost-effectiveness ratio's are calculated by dividing the difference between the mean costs of the two treatment groups by the difference in the mean effects of the two treatment groups. Cost-utility is based on the EuroQol and expressed in costs per quality adjusted life year. Cost-effectiveness and cost-utility ratios are estimated using boot-strapping techniques and graphically presented on cost-effectiveness and cost utility planes. Acceptability curves are also presented. Sensitivity analysis on the most important cost drivers are performed in order to assess the robustness of the results.

## Discussion

Several randomized controlled trials have been performed which dealt with continuity of care and described one or more interventions which are also conducted in our study (medication reconciliation, patient counselling and transfer of information on medication to primary care) [5,12-15]. Contrary to these studies we regarded a randomized design as not feasible, because previous experiences with pilot projects have shown that the COACH program contaminates usual care as residents and other healthcare providers learn from the COACH program. The program therefore influences prescribing behaviour and the organisation of care. Therefore, we have chosen for an observational before-after design including interrupted time series as the preferred alternative [39].

We expect this study to have several strengths. First, we have gained experience due to previous pilot projects and have been able to optimize the process such as accurate medication reconciliation and more structured patient counselling. We have also optimized documents such as the medication summary for the patient and the medication overview for the general practitioner and community pharmacist. Second, due to previous experiences pharmaceutical consultants are trained in recognizing drug-related problems. Third, in contrast to other studies we are also conducting a cost-effectiveness assessment. Finally, in this study we will estimate the effect of the COACH program in a multicultural population which will provide more insight in the effect of discharge counseling in ethnic minority patients.

This study also has some limitations. First, selection bias is possible as especially ethnic minority groups might not want to cooperate. This could also lead to failure to reach the recruitment target and hence could reduce the study's statistical power to detect differences in the primary outcome. Second, previous studies have shown mixed results. It is unknown which interventions are effective and how long the follow-up period should be. Nevertheless, we believe the comprehensive COACH program will be able to show effect on patient safety related outcomes. Finally, as it concerns a monocenter study this may limit generalisability.

Studies generally have shown the effect of discharge medication related interventions on reducing adverse events, medication errors and drug-related problems [5,12-15]. This study however, will be able to evaluate the clinical and cost impact of a comprehensive program on continuity of care. The possible impact of the COACH program on hospital readmissions will provide insight in the quality of care. The findings from this study will provide information of interest to many stakeholders, including patients, health care managers, policy makers and health care professionals.

## Competing interests

The authors declare that none of them have received honoraria, reimbursement or fees from any pharmaceutical companies.

## Authors' contributions

All authors are responsible for interpretation of the data and are involved with drafting and reviewing the manuscript. FKC, SB, PB and TE are responsible for study design, study implementation and data analysis. MT is responsible for the economic analysis. All authors have read and approved the final manuscript.

## Funding

The Dutch insurance company Agis gave financial support for this study.

## Pre-publication history

The pre-publication history for this paper can be accessed here:

http://www.biomedcentral.com/1472-6963/10/39/prepub
